# Signal Persistence of Bispectral Index and State Entropy during Surgical Procedure under Sedation

**DOI:** 10.1100/2012/272815

**Published:** 2012-02-02

**Authors:** Chanannait Paisansathan, Mukadder D. Ozcan, Qaiser S. Khan, Verna L. Baughman, Mehmet S. Ozcan

**Affiliations:** ^1^Department of Anesthesiology, University of Illinois at Chicago, 1740 West Taylor Street, Suite 3200W, Chicago, IL 60612, USA; ^2^Department of Anesthesiology, The University of Oklahoma Health Sciences Center, Oklahoma City, OK 73104, USA

## Abstract

*Introduction*. Bispectral index (BIS) and state entropy (SE) are prone to artifacts, especially due to electrocautery (EC). We compared the incidence of artifacts in BIS and SE during surgery under local anesthesia and sedation. *Methods*. 28 females undergoing breast surgery under local anesthesia and sedation were studied. Simultaneous BIS and SE measurements were recorded every 10 seconds. Artifact was defined as a failure of the device to display a numerical value while the electrodes remained appropriately attached to the patient's forehead. Ratio of artifact to good signal was compared between BIS and SE in the presence or absence of EC use. *Results*. 7679 data points were collected from 28 patients. Overall, artifact incidence was similar in BIS and SE (6.2% and 6.3%, resp.). In the presence of EC (1370 data points), BIS had significantly more artifact compared to SE (18.6% versus 6.4%, *P* < 0.0001). Without EC (6309 data points), BIS had significantly less artifact compared to SE (4.1% versus 7.3%, *P* < 0.0001). *Discussion*. BIS and SE were comparable for incidence of artifacts in patients under sedation. Use of EC lead to more artifact in BIS than SE. Conversely, BIS had fewer artifacts than SE when there was no EC use.

## 1. Introduction

Process analysis of the electroencephalogram (EEG) is increasingly used in anesthesiology for quantification of anesthetic drug effect. In the past decade, Bispectral index (BIS, Aspect Medical System, Newton, MA, USA), a complex parameter composed of a combination of time domain, frequency domain and higher order spectral analysis has been introduced [1]. Another concept to quantify electroencephalography is the entropy calculation. State entropy (SE) is an index that is computed using the irregularity, complexity, and unpredictability characteristics of the EEG signal [2]. Both monitors use frontal electroencephalogram (EEG), recorded through scalp surface electrodes, to compute an index that clinically correlates to a specific level of sedation.

Quality of any EEG-derived parameter is largely dependent on processing the raw signal, thus detecting and removing contamination by various artifacts commonly observed in the daily clinical routine. EEG monitoring using the scalp surface electrodes is prone to artifacts [3, 4]. The operating room (OR) is a challenging environment for artifact contamination. Besides, physiologic sources, such as electromyographic (EMG) and electrocardiographic (EKG) activity, numerous electrical devices used in the OR can cause EEG artifact. Among those, electrocautery (EC) is a well-recognized source of artifact [4].

Artifact recognition and rejection are incorporated into the algorithms of BIS and SE [1, 5]. Nevertheless, artifacts in both BIS and SE remain a problem as indicated by numerous case reports, studies, and review articles on this subject [6–10]. Observational studies have suggested that SE is more resistant to artifacts than BIS [5, 10]. However, many methodological details were not described in these studies, including a description of how the EC artifact was detected, duration of the EC activity, the proximity of the surgical site (i.e., EC stimulus) to the EEG electrodes, and a documentation of the coexisting EMG interference. Without accounting for these factors, it is difficult to conclude whether SE is more resistant to artifact interference than BIS, as suggested.

We hypothesized that SE and BIS would not be different in their resistance to EC artifact during surgery under sedation. To test our hypothesis, we simultaneously recorded SE and BIS (along with facial muscle EMG) in patients undergoing breast surgery under local anesthesia and sedation. Then we organized data points into two groups according to the presence or absence of EC interference. Finally, we analyzed and compared the incidence of artifact of SE and BIS in these two groups.

## 2. Methods

### 2.1. Study Population

After IRB approval and obtaining informed consent, 28 female patients who were older than 18 years and scheduled to have breast surgery with local infiltration anesthesia under sedation were enrolled in this study. Patients with a history of stroke, seizures, dementia, carotid stenosis, or any known brain pathology were excluded.

### 2.2. Study Protocol

All patients received 2 mg IV midazolam for anxiolysis upon entry to OR. BIS and entropy monitors were applied along with standard anesthesia monitoring to each patient before surgical sedation was started. After baseline vital signs were obtained, 50 to 100 *μ*g IV bolus of fentanyl was given and a propofol infusion was started as the surgical field was cleaned and draped. The propofol infusion was started at 25–50 *μ*g/kg/min and was titrated to attain a Ramsay sedation score of 3 to 4. Additional fentanyl was administered in 25 to 50 *μ*g increments as indicated for pain.

Both BIS and SE were monitored in all patients but only BIS was displayed on the OR monitor. Whether or not to use BIS to assist titration of sedation was left to anesthesiologists, who were not aware of the specifics of study protocol. They were only informed that this was a study comparing the intraoperative performances of BIS and entropy monitors. One of the investigators remained in the OR for the duration of the study and was solely responsible for collection of study data.

### 2.3. EEG Monitoring and Data Collection

BIS and entropy sensors were placed adjacent to each other on the patient's forehead and were continuously monitored and recorded every 5–30 s throughout the surgery. We used the entropy (GE Healthcare Finland, Helsinski, Finland) and the BIS-XP (Aspect Medical Systems, Newton, MA, USA). The EEG sampling rate was 256 Hz for BIS with a smooth rate of 10 seconds and 400 Hz for state entropy with time windows ranging from 15 to 60 seconds for SE calculation.

### 2.4. Definition of Artifact

In this study, artifact (for both BIS and SE) was defined as a failure of the module to display a numerical value, while the electrodes remained attached to the patient's forehead with acceptable impedance. Impedance checks of BIS and SE were automatically performed at the beginning of the monitoring and every 10 minutes thereafter. When the maximum acceptable limits of impedance (10 kΩ and 7.5 kΩ for BIS and SE, resp.) were exceeded, we followed the manufacturers' recommendation by applying digital pressure over the electrode with high impedance for 5 seconds. This was repeated until the impedance problem was resolved (indicated by the word “pass” appearing on the screen for the corresponding electrode). If the impedance problem remained unresolved for 2 minutes, the electrode strip was removed, skin was recleaned with a 70% isopropyl alcohol wipe, and a new electrode strip was applied. Such periods of artifact due to poor contact between the sensor and patient's skin (i.e., increased sensor impedance) were excluded from further analysis.

### 2.5. Determination of EC and Non-EC Periods

EC activity was detected by the audible signal of the diathermy unit and confirmed by its interference on the recorded EKG. During each surgical case, time periods were marked as having EC activity whenever the activity of the diathermy unit was heard. No distinction was made between the activation of cut and coagulate mode of EC activity. At a subsequent analysis, data sets collected during the EC activity periods were reviewed. From those data sets, only those with an EKG signal confirming interference were labeled as an “EC Period (EC period).” The interference was defined as either a complete loss of the EKG signal or disagreement of more than 20 points between pulse rate captured by the pulse oximeter and the heart rate recorded by the EKG during an EC activity. All other data points were labeled as “Non-EC Periods (non-EC period).”

### 2.6. Statistical Analysis

Proportions of artifact in BIS and SE data were compared using chi-squared test with Yates correction. A two-tailed *P* < 0.01 was considered significant. Recorded entropy and BIS data were not normally distributed (Kolmogorov-Smirnov-test, *P* < 0.005) and are reported as median and range. Normally distributed parametric data was tested with paired *t*-test, where *P* < 0.05 was considered significant. Nonparametric data was compared using Mann-Whitney test and *P* < 0.05 was considered significant.

## 3. Results

7679 sets of data were collected from 28 patients. Of those data sets, 1392 were marked as having EC activity. 1370 of the 1392 data sets were classified as EC period after the confirmation of the EC interference on the recorded EKG. The remaining 22 data sets without EKG interference were combined with the non-EC data sets (6309) and were classified as non-EC period.

**Figure 1:**
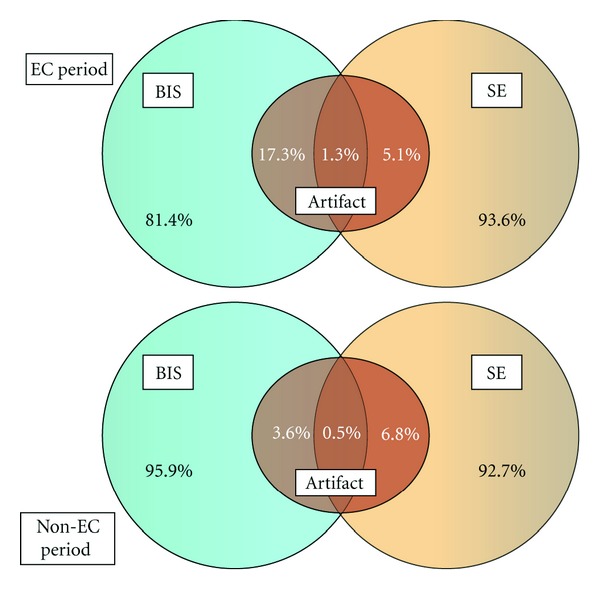
Bispectral index (BIS) and state entropy (SE) data with (top diagram) or without (bottom diagram) electrocautery (EC) use. The central set in each diagram represents data with artifact, whereas remaining set areas show good signal.

In EC period, a significantly higher proportion of BIS measurements (18.6%) had artifact compared to SE (6.4%). On the other hand, SE had significantly more artifact (7.3%) compared to BIS (4.1%) in non-EC period (Table 1). When data from both EC and non-EC periods were analyzed together, both modalities were similar in the incidence of artifacts (6.2 and 6.3% for BIS and SE, resp.; two-tailed *P* = 0.89). During both EC and non-EC periods, only a small proportion of artifacts were simultaneous in BIS and SE (Figure 1). Specifically, both modalities showed artifact in 1.3% and 0.5% of the data points during EC and non-EC periods, respectively. None of the patients required a replacement of the BIS or SE electrode strip for persistently high impedance.

Table 2 summarizes the parameters displayed by BIS and SE during EC and non-EC periods. Median SE values were similar during EC and non-EC periods. Median BIS was lower during the EC period, which was statistically significant. Facial EMG measured by BIS was similar in both the EC and non-EC periods. The BIS signal quality index (SQI) was significantly lower in the EC period. The BIS measurements with SQI < 50 were more than three times higher in EC period than those in the non-EC period.

## 4. Discussion

We found that SE and BIS were not different in their failure of index calculation during breast surgery under moderate sedation. However, there were more artifacts with BIS (18.6%) than SE (6.4%) during EC use, which is consistent with data recently reported by White et al. [10]. This may be due to the fact that the entropy module continuously measures power in the frequency range from 200 kHz to 1000 kHz. When this power exceeds a set threshold value, the EEG signal will be inspected. If the signal appears to be disturbed, the epoch is rejected from analysis. Conversely, BIS XP and later versions recognize artifact from signal asymmetry with a mirror image recorded from a supplementary electrode. BIS claims to improve the detection of signal contamination from EC or eye movement. Nonetheless, our result shows more artifacts in BIS module than SE when evaluating the effect of EC.

 Conversely, when EC is not in use, SE (7.3%) had more artifacts than BIS (4.1%). This indicates that SE is more susceptible than BIS to other sources of interference in the operating room in moderately sedated patients. Interestingly, it has been reported that entropy was more resistant to artifacts than BIS in brain-dead organ donors and patients undergoing cardiac surgery [5, 11]. The difference between our finding and this study series could be explained partly because we looked at the complete failure of the device rather than a falsely calculated index as reported in those studies. Secondly, we conducted the study in moderately sedated patients undergoing breast surgery as compared to brain-dead organ donors or general anesthesia in cardiac surgery. Patients in our study would have more spontaneous eye movements, blinks, and movement artifact. It is possible that artifact algorithm rejection for entropy that comprise of two-step method (stationary analysis and signal characteristic analysis) is less sensitive to above movement artifact compared to BIS artifact algorithm rejection in patient with moderately sedated undergoing surgery.

Artifact in electrophysiological measurements (such as the EEG) can be defined as the distortion of a signal, which interferes with or obscures the interpretation of EEG study and potentially lead to wrong management. Source of artifacts in the clinical setting such as in the operating room and ICU can come from external and internal factors. External factors commonly encountered in the operating room include EC and other electrical generating signal devices such as monitors, fluid warmers, and blanket warmer devices. Internal factors include electromyography (facial EMG), eye movement, ballistocardiography (small rhythmic movements of the head induced by cardiac contraction and ejection of blood through the vessels), and electrocardiography (EKG). As a result, EEG monitoring devices commonly used in the clinical setting (e.g., entropy and BIS) need to reliably distinguish EEG signal from contamination. In this study, we limited the definition of artifact to a failure of the device to generate an index. Total failure of index calculation is important for considering the usefulness of certain devices in different clinical setting. In the operating room, moderate sedation and the use of EC are common practices. Failure of index calculation due to such interferences may potentially lead to inadequacy of anesthetic or sedation level during painful stimulation, patient's awareness and discomfort, sudden movement, and dissatisfaction during the procedure. Until now, a comparison of SE and BIS in their failure to calculate an index has not been reported in moderately sedated patients. However, by limiting the artifact to inability of the device to calculate an index, we might be missing other instances of artifact where a falsely high or low index is generated.

 The BIS is the result of a proprietary combination of three main variables [1]. One of the important variables is the beta ratio, which dominates the calculation during sedation. It is calculated on a frequency range that overlaps EMG frequencies in the 30–47 Hz intervals. Since our result showed no difference in EMG activity during EC and non-EC periods, it is less likely for facial muscle activity to be a cause for artifact in our study.

 We defined EC activity as an activation of the diathermy unit along with a related interference on the EKG signal. This was to identify the data points where the EC unit is actually in contact with the patient. This approach has some limitations, for example, EC activity may cause an artifact on BIS and SE, but not on EKG. However, when we analyzed our data, only 1.6% of data points in EC period were without a concomitant EKG interference. Therefore, this is unlikely to affect our results.

 In conclusion, our study indicates that SE is more resistant to artifact compared to BIS during EC use, while BIS is more resistant to other artifacts that can be encountered during surgery in moderately sedated patients. Both modules still have failure of index calculation despite adequate impedance. However, artifact interference may be different with a deeper level of anesthesia. In the clinical setting requiring light to moderate sedation with continuous EEG monitoring without EC, such as in the intensive care unit, BIS might carry less risk of index failure from artifacts compared to SE.

## Figures and Tables

**Table 1:** Number of data points of SE and BIS with artifact or good signal.

	EC period (*n* = 1370)		Non-EC period (*n* = 6309)		All periods (*n* = 7679)	
	BIS	SE	BIS	SE	BIS	SE
Artifact	255	88	223	395	478	483
Good signal	1115	1282	6086	5914	7201	7196
Artifact ratio	18.6%*	6.4%	4.1%	7.3%*	6.2%	6.3%

Artifact ratio: number of artifacts/number of all signals, EC: electrocautery, BIS: bispectral index, SE: state entropy, comparisons between BIS and SE for each period, *two-tailed *P* < 0.0001, chi-square with Yates correction.

**Table 2:** Comparison of SE and BIS parameters during EC and non-EC periods.

	EC period	Non-EC period	*P*
SE	77 (46–89)	80 (45–89)	0.09
BIS	70 (47–83)	75 (42–86)	<0.0001*
EMG (dB)	41 ± 9	42 ± 9	0.09
SQI < 50 (%)	40.7%	12.3%	<0.0001*
SQI (Mean ± SD)	59 ± 30	78 ± 24	<0.0001*

EC: electrocautery, SE: state entropy, BIS: bispectral Index, EMG: electromyogram, SQI: signal quality index. SE and BIS are expressed as median (10% quartile–90% quartile). Comparisons for SE, BIS, EMG, and SQI between EC and non-EC periods were made using Mann-Whitney test. Comparison for SQI < 50 between EC and non-EC periods was made using chi-square test with Yates correction.
